# Assessment of physiological signs associated with COVID-19 measured using wearable devices

**DOI:** 10.1038/s41746-020-00363-7

**Published:** 2020-11-30

**Authors:** Aravind Natarajan, Hao-Wei Su, Conor Heneghan

**Affiliations:** Fitbit Research, 199 Fremont St, Floor 14, San Francisco, CA 94105 USA

**Keywords:** Health care, Health policy

## Abstract

Respiration rate, heart rate, and heart rate variability (HRV) are some health metrics that are easily measured by consumer devices, which can potentially provide early signs of illness. Furthermore, mobile applications that accompany wearable devices can be used to collect relevant self-reported symptoms and demographic data. This makes consumer devices a valuable tool in the fight against the COVID-19 pandemic. Data on 2745 subjects diagnosed with COVID-19 (active infection, PCR test) were collected from May 21 to September 11, 2020, consisting of PCR positive tests conducted between February 16 and September 9. Considering male (female) participants, 11.9% (11.2%) of the participants were asymptomatic, 48.3% (47.8%) recovered at home by themselves, 29.7% (33.7%) recovered at home with the help of someone else, 9.3% (6.6%) required hospitalization without ventilation, and 0.5% (0.4%) required ventilation. There were a total of 21 symptoms reported, and the prevalence of symptoms varies by sex. Fever was present in 59.4% of male subjects and in 52% of female subjects. Based on self-reported symptoms alone, we obtained an AUC of 0.82 ± 0.017 for the prediction of the need for hospitalization. Based on physiological signs, we obtained an AUC of 0.77 ± 0.018 for the prediction of illness on a specific day. Respiration rate and heart rate are typically elevated by illness, while HRV is decreased. Measuring these metrics, taken in conjunction with molecular-based diagnostics, may lead to better early detection and monitoring of COVID-19.

## Introduction

The year 2020 has seen the emergence of a global pandemic caused by the severe acute respiratory syndrome coronavirus 2 (SARS-CoV-2) virus. The disease caused by this virus typically presents as a lower respiratory infection, though many atypical presentations have been reported. This has caused a major health challenge globally due to the apparent high transmissibility of this virus in a previously unexposed population. Of particular concern is that the primary mechanisms by which the disease is transmitted are still somewhat under debate (e.g., the importance of airborne transmission)^1^, and the potential for infection by asymptomatic and pre-symptomatic patients (see for e.g., the discussion in Oran and Topol^2^). The disease is highly contagious, with transmission possible 2.3 days prior to the onset of symptoms, and peaking 0.7 days prior to the onset of symptoms according to one model^3^. As a result, a great deal of effort is underway to potentially diagnose COVID-19 early.

The popularity and widespread availability of consumer wearable devices has made possible the use of health metrics such as respiration rate, heart rate, heart rate variability (HRV), sleep, steps, etc. in order to predict the onset of COVID-19 or similar illnesses. A 1 °C rise in body temperature can increase heart rate by 8.5 beats per minute (b.p.m.) on average^4^. Measuring the resting heart rate, or heart rate during sleep can therefore be a useful diagnostic tool. Similarly, the respiration rate is elevated when patients present with a fever^5^. HRV is the variability in the time between successive heart beats (the time between successive heart beats is called the “RR interval”), and is a valuable, non-invasive probe of the autonomic nervous system^6–8^. Lowered values are indicative of increased mortality^9^, and may provide early diagnosis of infection^10^. A study of HRV in critically ill COVID-19 patients showed that the approximate entropy and the sample entropy were decreased in COVID-19 patients compared to critically ill sepsis patients^11^.

Zhu et al.^12^ studied heart rate, activity, and sleep data collected from Huami wearable devices to potentially identify outbreaks of COVID-19, and concluded that at a population level an anomaly detection algorithm provided correlation with the measured infection rate. Menni et al.^13^ analyzed symptoms reported through a smartphone app and developed a model to predict the likelihood of COVID-19 based on the symptoms. Marinsek et al.^14^ studied data from Fitbit devices as a means for early detection and management of COVID-19. Miller et al.^15^ used the respiration rate obtained from Whoop devices to detect COVID-19. Mishra et al.^16^ analyzed heart rate, steps, and sleep data collected from Fitbit devices to identify the onset of COVID-19.

In this paper, we consider the correlation between changes in physiological signs related to respiration rate, heart rate and HRV, and the corresponding presence of diseases assessed both through confirmed laboratory testing and self-reported symptoms and the time-course of the disease. We show that it is possible to use changes in these physiological metrics to detect illness, and provide estimates of sensitivity and specificity. In addition, given the reporting of symptoms by study participants, we provide an estimate of predicted disease severity based solely on symptoms.

## Results

### Prediction of hospitalization based on symptoms

**Fig. 1:**
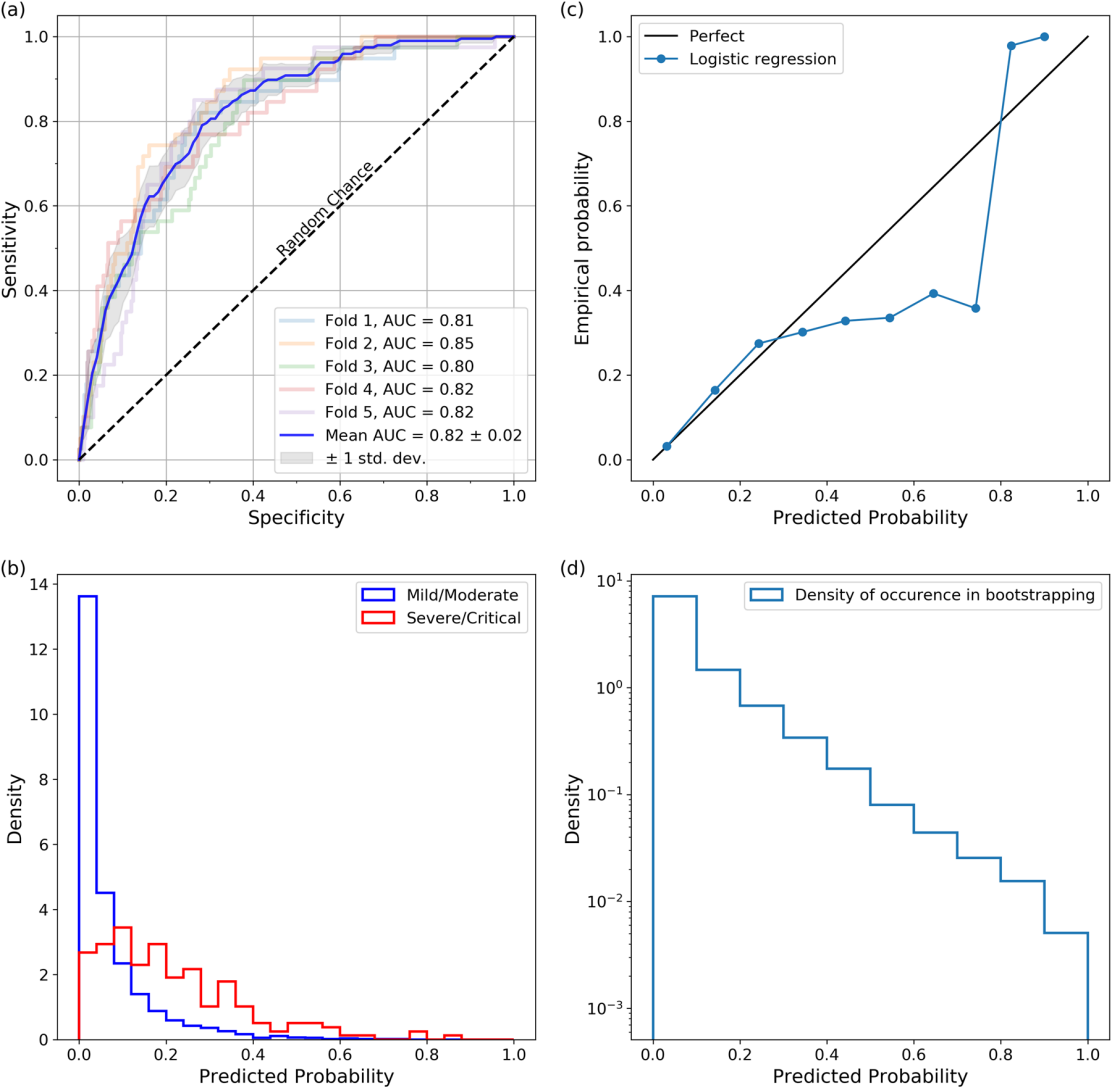
Predicting the need for hospitalization given the symptoms. **a** The AUC averaged over five folds is 0.82 ± 0.02. **b** Predicted probability distribution for mild/moderate versus severe/critical cases (area normalized to 1). The class imbalance influences the classifier probabilities. **c** Reliability plot done by bootstrapping the logistic regression with different 80–20% train-test splits repeated 20,000 times. The predicted probability is more accurate below 25% which is where most samples are located. **d** Normalized distribution of the bootstrapped predictive probability.

We first describe the results of the logistic regression classifier for the prediction of hospitalization based on the symptoms presented. Figure 1a shows the ROC curve where “true positive” indicates a prediction of hospitalization for an individual who indeed required hospitalization. Averaged over five folds, the area under ROC (AUC) is 0.82 ± 0.017. Figure 1b shows the distribution of classifier probabilities for mild/moderate cases (who did not require hospitalization) and for severe/critical cases (subjects who were hospitalized). Note that the classifier probabilities are not true probabilities in the frequentist sense, and are influenced by the class imbalance. Figure 1c shows the reliability plot done by bootstrapping the logistic regression with different 80–20% train-test splits repeated 20,000 times. The predicted probability is more accurate below 25% which is where most samples are located. Figure 1d shows the normalized distribution of the bootstrapped predictive probability. The probability of the need for hospitalization *p* may be expressed as:

1 $$\begin{array}{rcl}z&=&\alpha + \mathop {\sum}\limits_{i}{w}_{i}{s}_{i}\\ p&=&\frac{1}{1\,+\,{e}^{-z}},\end{array}$$

where *α* = −3.631, *s*_*i*_ is a symptom (1 if the symptom is present, and 0 otherwise), and *w*_*i*_ is the weight corresponding to symptom *s*_*i*_. The weights for the various symptoms are shown in Table 1. We rescale the two continuous variables (age and BMI) as follows:

2 $${x}_{s}=\frac{x-{\mu }_{x}}{{\sigma }_{x}},$$

where *x*_*s*_ is the scaled version and *x* stands for age or BMI; *μ*_*x*_ and *σ*_*x*_ are the respective mean and standard deviation, which are (40.75 years, 12.31 years) for age and (30.77, 7.49) for BMI. For the sex, 1 indicates male and 0 indicates female.

**Table 1:** Predicting the need for hospitalization: importance of symptoms.

Symptom	Weight
Shortness of breath	1.325
Vomiting	0.891
Confusion	0.822
Fever	0.645
Loss of appetite	0.563
Age (scaled)	0.456
Swelling in the fingers and toes	0.379
BMI (scaled)	0.376
Chest pain	0.303
Cough	0.167
Sex (male: yes/no)	0.117
Diarrhea	0.082
Rash	0.060
Chills	0.042
Body aches	−0.008
Decrease in taste and smell	−0.057
Hoarse voice	−0.082
Eye pain	−0.205
Neck pain	−0.267
Stomach ache	−0.283
Fatigue	−0.293
Sore throat	−0.398
Head ache	−0.458
Congestion	−0.750

### Change in biometrics and classification of illness

**Fig. 2:**
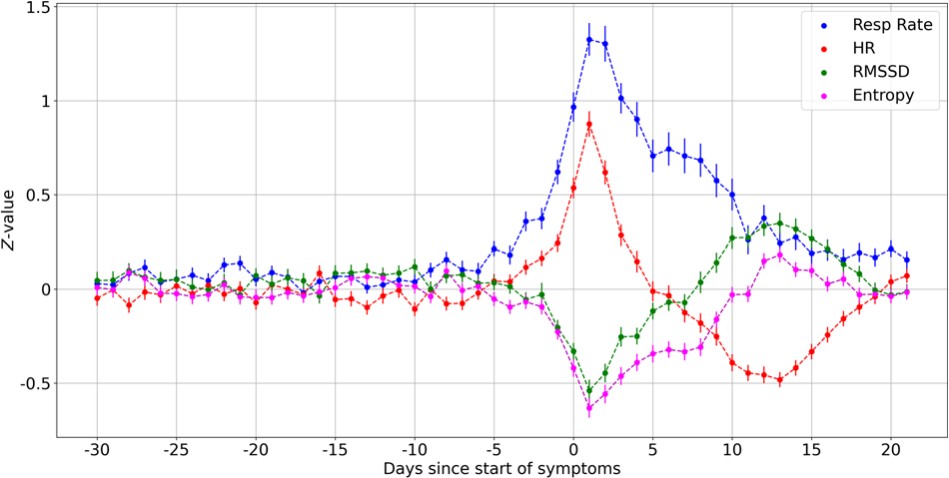
Variation of metrics with day. Shown are the *Z*-scores for respiration rate, heart rate, RMSSD, and entropy. Day 0 (*D*_0_) represents the start of symptoms. The respiration rate and heart rate are elevated during times of sickness, while the RMSSD and entropy are decreased. These metrics may change a few days prior to the start of symptoms. The heart rate decreases on average, following day *D*_+7_, and returning to the base value by day *D*_+21_. The HRV metrics are slightly elevated on average during this period. We did not notice a decrease in respiration rate during this phase. Error bars represent the standard error of the mean.

Let us now consider the problem of determining whether an individual is sick or healthy given the physiological metrics. Figure 2 shows the average *Z*-scores of symptomatic individuals for the respiration rate, heart rate, root mean square of successive differences (RMSSD), and entropy as a function of day, where day *D*_0_ represents the start of symptoms. The error bars represent the standard error of the mean. The respiration rate shows the largest effect and also takes the longest time to return to its base value. It is interesting to note that the heart rate decreases on average, following day *D*_+7_, and returning to the base value by day *D*_+21_. The HRV metrics, on the other hand, are slightly elevated on average during this period. We did not notice a decrease in respiration rate, on average, during this phase.

The neural network was trained to predict whether an individual is sick on any specific day given the *Z*-scores for respiration rate, heart rate, RMSSD, and entropy for that day and the preceding 4 days. Let *k* be the filter size for the one-dimensional convolution, and let *m* be the number of filters. Let *N*_1_ and *N*_2_ be the number of neurons in the first and second dense layers, and let *α* be the drop out rate, i.e., the rate at which input units are set to zero (https://keras.io/api/layers/regularization_layers/dropout/).

We set reasonable ranges for the various hyper-parameters, and computed the largest AUC using the cross-validation set, from 500 random parameter choices. The best parameters were found to be *k* = 12, *m* = 64, *N*_1_ = 16, *N*_2_ = 32, *α* = 0.4. We obtained the best performance considering the 14 days from *D*_−21_ to day *D*_−8_ to be negative class examples, and the 7 days from *D*_+1_ to day *D*_+7_ to be positive class examples. The days from *D*_−7_ to *D*_0_ are discarded.

**Fig. 3:**
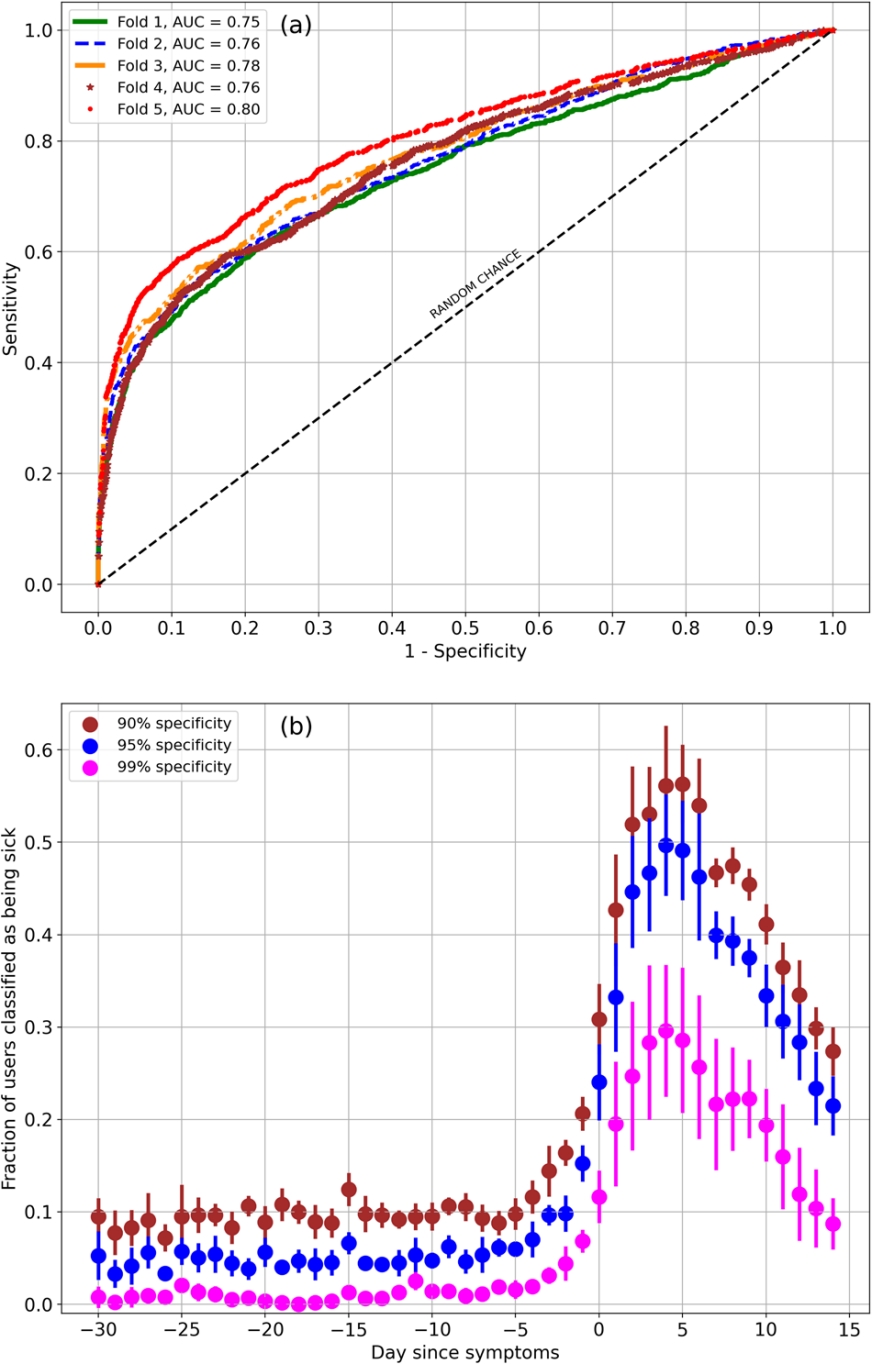
Classifier performance. Predicting sickness given the physiological signs: **a** with five-fold validation, the AUC is 0.77 ± 0.018. Data from day −21 to day −8 were treated as negative cases, while data from day +1 to day +7 were assumed positive. Data from day −7 to day 0 were ignored. Day 0 was the day when symptoms were reported. The sensitivity is 0.259 ± 0.059 at 99% specificity, 0.437 ± 0.037 at 95% specificity, and 0.513 ± 0.034 at 90% specificity. **b** The fraction of users predicted positive on specific days, from day −30 to day +14, for specificity requirements of 99% (magenta), 95% (blue), and 90% (brown). Errors bars are 1 standard deviation.

The sensitivity/specificity plot (ROC curve) measured on the test dataset is shown in Fig. 3a. The AUC computed using data in the test set, and averaged over five folds is 0.77 ± 0.018. The sensitivity averaged over five folds at 99% specificity is 0.259 ± 0.059. The sensitivity at 95% specificity is 0.437 ± 0.037, and at 90% specificity, the sensitivity is 0.513 ± 0.034. Figure 3b shows the fraction of users who are predicted as sick on a specific day, from day *D*_−30_ to day *D*_+14_ (recall that as before, day *D*_0_ is the day when symptoms are reported), for three cases: (i) 99% specificity (magenta), (ii) 95% specificity (blue), and (iii) 90% specificity (brown). Averaged over the 21-day period from day *D*_−30_ to day *D*_−10_, the mean and standard deviation for the false-positive rate (assuming participants are all healthy during this period) for the 99% specificity model is 0.0085 ± 0.0062. For the 95% specificity model, the estimated false-positive rate over the same time period is 0.047 ± 0.0081. For the 90% specificity scenario, the false-positive rate is 0.094 ± 0.011. On day *D*_−1_, the fraction of users who are predicted as sick for the 99%, 95%, and the 90% specificity models are, respectively, 6.84 ± 1.22%, 15.25 ± 1.95%, and 20.63 ± 1.84%. On day *D*_+4_ for the same three models, the fraction of users who are predicted as sick are, respectively, 29.60 ± 7.13%, 49.68 ± 5.48%, and 56.12 ± 6.52%.

The prediction rate is higher for participants who present with a fever (for the Fold #5 classifier, the rate for the prediction of illness is about 12.8% higher averaged over days *D*_0_–*D*_6_). The prediction rate is also slightly higher for males compared to females. We attribute this difference to the fact that males are more likely to present with a fever compared to females (59.4% for males compared to 52.0% for females). Males are also more likely to require hospitalization (9.8% hospitalization rate for males compared to 7.0% for females). Males are also slightly older on average (43.8 ± 13.2 years for males compared to 40.4 ± 12.2 years for females).

### Classifier performance on healthy individuals

To estimate the false-positive rate on healthy participants, we selected 300 participants at random (89 male, 211 female, mean (std dev) of age = 45.1 years (13.3 years), mean (std dev) of BMI = 29.0 (7.2)). We consider a participant to be healthy if they did not report any symptoms and did not report taking a COVID-19 test or an influenza test. Each healthy participant had on average 142.2 days of data with a standard deviation of 51.6 days. With the classifier set to 95% specificity, there were 1722 positive predictions and a total of 42,655 user-days, yielding a false-positive rate of 4.04%. The mean false-positive rate computed per individual was 4.17% with a standard deviation of 4.33%. The median false-positive rate per individual was 2.82% and 90% of users had a false positive rate below 9.4%.

To estimate how correlated the positive predictions are, we calculated the probability of the occurrence of a contiguous sequence of *s* positive predictions. The case *s* = 1, i.e., a single positive prediction with no immediately succeeding or preceding positive prediction, appears 60.6% of the time. The probability of occurrence for *s* = 2, 3, 4, 5 are, respectively, 14.3%, 7.6%, 5.3%, and 6.6%. Let us compare this to a simulation of random positive predictions. For each individual, we estimated the positive rate *p* as the fraction of total predictions that are positive. We then set the prediction for any given day to be either sick with a probability *p* or healthy with a probability 1−*p*, and made predictions drawn from a binomial distribution, for each day of data for that participant. From this randomly assigned data, we computed the probability of a contiguous sequence of *s* positive predictions, averaged over 100 randomizations. For the random assignment, the case *s* = 1 occurs 92.3% of the time. The sequences *s* = 2, 3, 4, 5 occur with probabilities of 6.8%, 0.72%, 0.13% and 0.032%. Comparing the probabilities for the random distribution to the probabilities computed from the true data, it appears that the predictions are correlated, which raises the possibility that some of the individuals were ill on days when the predictions were made. It is also possible that our algorithm creates a correlation between days since it analyzes 5 days, and a large value on a certain day might affect the prediction on succeeding days. We note here that it is certainly possible for our “healthy” participants to have contracted a minor illness without presenting symptoms, during the course of 142.2 ± 51.6 days.

## Discussion

In this article, we analyzed data on 2745 subjects diagnosed with COVID-19 using the active infection PCR swab test with test dates ranging from February 16 to September 9, 2020. All subjects wore Fitbit devices and resided in the United States or Canada. With a total of 30,534 PCR tests, the overall positivity rate for PCR tests was 9.0%. Considering male (female) participants,11.9% (11.2%) of participants were asymptomatic, 48.3% (47.8%) recovered at home by themselves, 29.7% (33.7%) recovered at home with the help of someone else, 9.3% (6.6%) required hospitalization without ventilation, and 0.5% (0.4%) required ventilation. Fatigue was the most common symptom, present in 66.1% of males and 76.1% of females. Fever was present in 59.4% of males and 52% of females. The prevalence of some symptoms can be significantly different for male and female subjects.

The duration of symptoms depends on the severity: mild cases show a median duration of 11 days, while moderate cases have a median duration of 16 days. The median duration for cases that required hospitalization was found to be 25 days with a large spread. For mild, moderate, and severe/critical cases, the fraction of participants with duration of symptoms exceeding 60 days in our survey was found to be 3.9%, 6.4%, and 16%. We provided a simple formula to estimate the need for hospitalization given the symptoms, age, sex, and BMI. Shortness of breath is highly indicative of the need for hospitalization, while sore throat and stomach ache were the least likely. Rather surprisingly, gastrointestinal symptoms such as vomiting and loss of appetite were indicative of severe illness. Among demographic information, being older and having a high BMI show higher likelihoods for hospitalization.

We showed that respiration rate, heart rate, and HRV are useful indicators of the onset of illness. We trained a convolutional neural network to predict illness on any specific day given health metrics for that day and the preceding 4 days. To train the classifier, data from day *D*_−21_ to day *D*_−8_ were considered “healthy”, while data from day *D*_+1_ to day *D*_+7_ were labeled “sick”, where *D*_0_ is the date when symptoms present. For the purpose of training, data from *D*_−7_ to *D*_0_ were not considered. The AUC averaged over five randomizations was found to be 0.77 ± 0.018. The sensitivity at 99% specificity is 0.259 ± 0.059. At 95% specificity, the sensitivity is 0.437 ± 0.037, and at 90% specificity, the sensitivity is 0.513 ± 0.034. We then used the classifier to make a prediction for each date when data were available. With 90% specificity, the classifier can detect 43% of cases on day *D*_+1_. The classifier can detect 31% of cases on day *D*_0_, and 21% of cases on day *D*_−1_. When set to 95% specificity, we can detect 33% of cases on day *D*_+1_, 24% of cases on day *D*_0_, and 15% on day *D*_−1_. At 99% specificity, we can detect 20% of cases on day *D*_+1_, 12% of cases on day *D*_0_, and 6.8% on day *D*_−1_.

It is interesting to examine how this classifier may be applied as a screening tool for which positive predictive value and negative predictive value are more useful than sensitivity and specificity. Let us examine how the classifier works in an environment wherein the disease prevalence is similar to the situation in New York state in April 2020. From April 8 to April 21, the mean (standard deviation) number of confirmed COVID-19 cases per day in New York state was 8159 (2183)^17^ while the population of New York state is estimated to be 19.44 Million (https://worldpopulationreview.com/states/new-york-population), yielding a rate of 0.042% of the state population, for confirmed cases per day. The true prevalence of COVID-19 is expected to be much higher than this, since some individuals might not have had access to testing, some results might have been false negatives, and some people were likely asymptomatic and unaware of being ill. A study of COVID-19 antibodies implies a seroprevalence of 33.6% in New York state, considering data up to July 2020 (ref. ^18^). The total number of COVID-19 cases up to July 31, 2020, is estimated to be 419,723 (ref. ^17^), which is 2.159% of the state population. Comparing with the seroprevalence, and assuming antibodies result from all infected cases, we infer a factor of 15.56 COVID-19 cases per detected case. We therefore estimate a disease prevalence of 0.6535% per day for COVID-19 in New York state around the middle of April 2020. Setting the classifier specificity to 99%, the sensitivity is 0.259, and assuming a disease prevalence of 0.6535% per day, we find the positive predictive value is 14.55%, i.e., 1 in every 6.87 positive predictions is a true positive. The negative predictive value is 99.51%, and thus a negative result is highly predictive that the individual is healthy. Hence in a high case-rate region/period of time, individuals who receive a positive result should take precautions, or follow-up with a diagnostic test.

This study has multiple limitations which may confound some of its findings. The survey participants were all Fitbit users who may not represent the general US and Canadian population, and were all self-selecting in responding to the survey. Participants were asked to self-recall the start-date and end-date of any symptoms they experienced which may be quite unreliable. Participants may also confuse active (PCR) tests with serological (antibody) tests. In order to simplify the survey, we did not ask for a breakdown of symptom presentation and severity through out the time-course of the disease. It is also not possible to claim that our classifier can distinguish between COVID-19 and other respiratory illnesses such as influenza (our classifier predicted a positive result in many cases of influenza). For the prediction of the need for hospitalization, our data consisted of 196 positive cases and 2179 negative cases, so it is possible that our results are potentially affected by the small number of positive cases, as well as by the class imbalance. Nevertheless, we believe this survey provides an important scientific contribution by suggesting (a) hospitalization risk can be calculated from self-reported symptoms, and (b) relevant and predictive physiological signs related to COVID-19 may be detected by consumer wearable devices.

## Methods

### Data collection

Fitbit is a large manufacturer of wearable devices since 2007, and has a large established base of users (over 30 million as of 2020). A significant percentage of its devices are configured to measure heart rate, and the underlying interbeat intervals (RR) that characterize HRV. The Fitbit app provides a convenient user-facing app that can be configured to present user-facing questions, and to reliably capture responses in a secure and scalable way. In this study, active Fitbit users in the USA and Canada were invited to participate in a survey of whether they have experienced COVID-19 or similar infections, whether they had been tested, and to report on symptoms they experienced. They could also optionally provide additional demographic data such as age, sex, body mass index, and relevant background medical information such as underlying conditions such as diabetes, coronary arterial disease, or hypertension. While the researcher hypothesis was that metrics that could be generated from heart rate were likely to be most predictive of infectious disease, we did not restrict the survey to only Fitbit users with heart rate enabled devices (in practice, 95% of survey respondents had heart rate enabled devices).

**Fig. 4:**
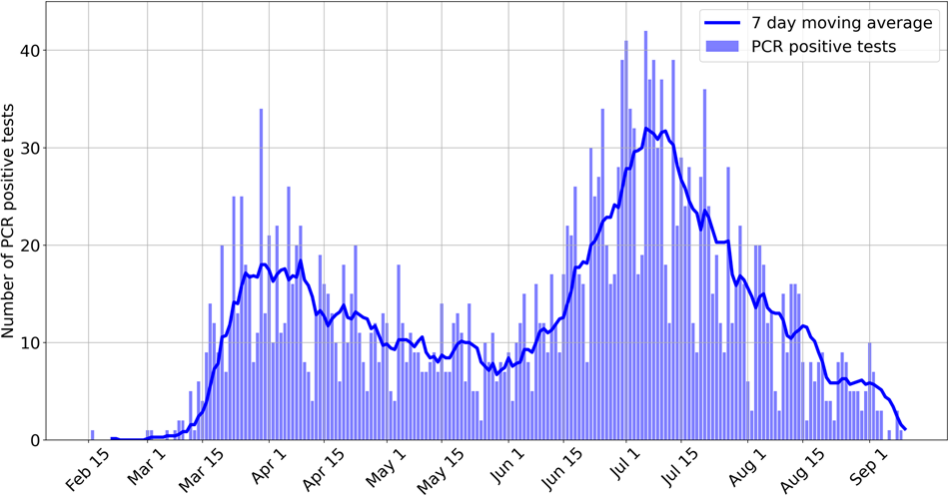
Distribution of PCR positive tests. Distribution of the number of PCR positive tests per day in our survey, from February 16 to September 9, along with the 7-day moving average. The distribution matches well with the reported cases. We stopped actively recruiting participants around the middle of July, which likely resulted in lower participation thereafter.

The survey and associated marketing and recruitment materials were approved by an Institutional Review Board (Advarra) and from May 21, 2020, the survey was available for completion by Fitbit users in the USA and Canada. The participants provided written informed consent for their data to be used in this study. The data presented here represent the analysis of survey results collected up to September 11, 2020. Figure 4 shows the number of PCR positive test results reported from February 16 to September 9, along with the 7-day moving average. Fitting the data to a bi-modal gaussian, we find the first peak on ≈April 9 with a standard deviation of 20.4 days, and a second peak on ≈July 7 with a standard deviation of 24.5 days.

The survey contained the following questions in relation to COVID-19 and other likely confounding infectious diseases such as influenza, urinary tract infections, etc.—(a) have you been tested for COVID-19 (with separate sections provided for tests for active infection versus serological tests for previous infection), (b) what were the symptoms experienced and the dates of onset and disappearance of the symptoms, (c) were you tested for other infectious diseases such as influenza, strep throat, etc.

Table 2 shows the overall breakdown of the survey responses as well as providing summary demographic information on the survey respondents. The age distribution of survey respondents was very similar to Fitbit users’ overall age distribution. Survey respondents skewed slightly more female than the corresponding figure for the overall general Fitbit population.

**Table 2:** Breakdown of overall survey results by summary demographics and test responses.

*n* = 187, 573 completed survey		
**Overall demographics**		
Age (mean ± std)	43.0 ± 13.9	
Sex (M/F/Other)	50,568 / 134,891 / 2114	
**Active COVID**	*n*	% of participants
**Infection tests (PCR)**		
Tests reported	30,534	16.28%
Positive	2745	0.15%
Negative	25,445	13.57%
Awaiting results/unknown	2343	0.12%
**Serology tests**	*n*	% of participants
Tests reported	13,550	7.22%
Positive	1117	0.60%
Negative	11,567	6.17%
Awaiting results/unknown	864	0.46%
**Flu tests**	*n*	% of participants
Tests reported	3894	2.08%
Positive tests	794	0.42%
Negative	3061	1.63%
Awaiting results/unknown	38	0.02%
**Other tested infections**	*n*	% of participants
Tests reported	1691	0.90%

Table 3 describes the self-reported major co-morbidities of the participants who reported a positive diagnosis of COVID-19, either through a confirmed PCR test or a serological test (we assume that the vast majority of tests for active COVID-19 infection were done using PCR-based techniques). Note that participants could also decline to answer this question, so these numbers are only indicative of general trends in the disease population.

**Table 3:** Self-reported health characteristics of participants who reported either a positive PCR test or a positive serological test; note that some participants may have received both.

Characteristics of COVID-19 participants		
Age (mean ± std)	41.2 ± 12.8 years	
Sex (M/F/Other)	834 / 2691 / 21	
**Self reported health**	*n*	%
Hypertension	636	17.9%
Asthma	635	17.9%
Diabetes	252	7.1%
Coronary arterial disease	51	1.4%
Stroke	32	0.9%
Chronic lung disease	43	1.4%
Chronic kidney disease	36	1.0%
Congestive heart failure	23	0.6%

In order to assess some metric of disease severity, we took the approach of asking about the person’s treatment rather than through quantification of symptoms. The options provided to a survey participant were:

- 1) I didn’t experience symptoms.
- 2) I self-treated alone.
- 3) I self-treated with someone’s help.
- 4) I required hospitalization without ventilation support.
- 5) I required ventilation.
- Prefer not to say.

**Fig. 5:**
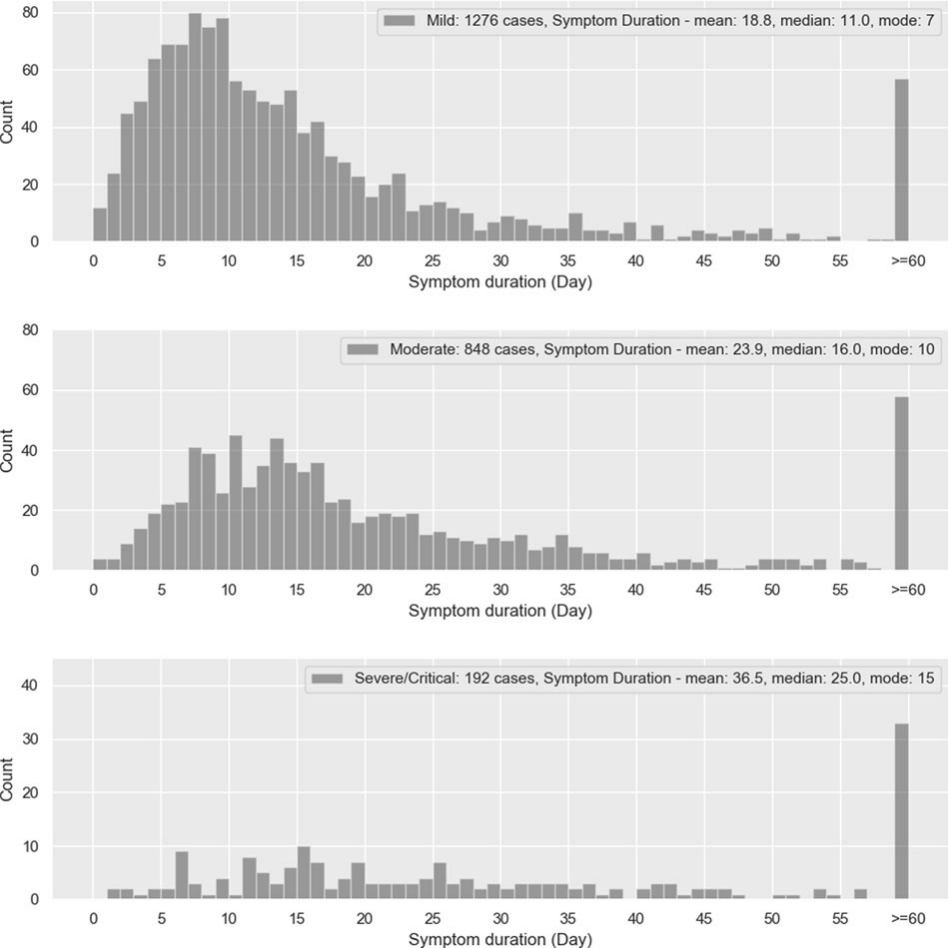
Duration of symptoms. Distribution of symptom duration for mild, moderate, and severe/critical cases. The median symptom duration is 11 days for mild cases, 16 days for moderate cases, and 25 days for severe/critical cases. For mild, moderate, and severe/critical cases, the fraction of participants with duration of symptoms exceeding 60 days is found to be 3.9%, 6.4%, and 16%, respectively.

We consider 1 as asymptomatic, 2 is assigned to category “mild”, 3 is assigned to “moderate”, 4 is “severe”, and 5 is “critical”. Considering male (female) participants, we find that 11.9% (11.2%) of participants were asymptomatic, 48.3% (47.8%) had mild symptoms, 29.7% (33.7%) were moderate, 9.3% (6.6%) severe, and 0.5% (0.4%) critical. Those with mild symptoms recovered sooner than those with moderate or severe symptoms. The distribution in the duration of symptoms is shown in Fig. 5. There were 21 symptoms that were reported. The distribution of symptoms by severity, for male and female participants, is tabulated in Table 4. We do not have information regarding the timing of symptoms, i.e., we do not know which symptoms appeared first.

**Table 4:** Prevalence of symptoms.

Symptom	All (%)	Asx.	Mild(%)	Mod(%)	Sev/crit(%)
	Male (female)	–	Male (female)	Male (female)	Male (female)
Fatigue	66.1 (76.1)	–	69.9 (80.5)	82.9 (92.3)	76.8 (89.1)
Headache	53.7 (71.4)	–	57.6 (76.0)	68.2 (86.9)	57.1 (78.3)
Body ache	58.4 (64.4)	–	59.4 (64.8)	77.1 (81.4)	69.6 (81.9)
Decrease in taste & smell	50.2 (64.9)	–	54.3 (69.6)	62.4 (77.1)	53.6 (76.1)
Cough	55.2 (59.9)	–	50.0 (59.1)	78.8 (76.2)	76.8 (81.9)
Fever	59.4 (52.0)	–	57.6 (48.0)	78.2 (68.5)	83.9 (80.4)
Chills	51.0 (51.1)	–	50.4 (47.0)	68.8 (68.6)	64.3 (73.9)
Congestion	36.9 (53.0)	–	42.8 (60.8)	48.2 (61.5)	19.6 (43.5)
Loss of appetite	39.3 (48.0)	–	36.6 (44.6)	51.8 (61.8)	62.5 (79.0)
Shortness of breath	37.2 (46.1)	–	25.4 (38.1)	57.6 (64.4)	80.4 (84.1)
Sore throat	30.1 (45.7)	–	30.1 (49.6)	41.8 (56.3)	32.1 (39.1)
Diarrhea	32.7 (42.0)	–	30.8 (38.5)	44.7 (57.3)	44.6 (58.0)
Chest pain	30.2 (41.9)	–	27.2 (35.4)	42.9 (59.7)	44.6 (65.2)
Neck pain	14.3 (26.3)	–	15.2 (23.9)	18.8 (37.3)	14.3 (30.4)
Hoarse voice	15.0 (23.5)	–	13.0 (22.0)	20.0 (32.0)	28.6 (27.5)
Stomach ache	10.1 (22.9)	–	10.5 (20.1)	13.5 (33.2)	10.7 (28.3)
Eye pain	13.8 (21.0)	–	15.2 (19.9)	15.9 (28.7)	17.9 (23.2)
Confusion	15.2 (18.0)	–	10.9 (13.5)	23.5 (25.8)	30.4 (38.4)
Vomiting	5.2 (10.6)	–	4.3 (7.2)	6.5 (14.6)	12.5 (29.0)
Rash	4.7 (8.3)	–	5.8 (6.7)	5.9 (11.6)	1.8 (15.9)
Swelling in the fingers & toes	2.1 (6.4)	–	1.8 (4.2)	2.4 (9.5)	5.4 (14.5)

### Making predictions of hospitalization based on symptoms

Using the symptoms along with demographics as input features, we trained a logistic regression classifier to predict the need for hospitalization. We only considered symptomatic individuals for this analysis. We trained a logistic regression model to predict the need for hospitalization with five-fold validation (80% of the data used to train, 20% used to test), using the symptoms as input features, along with the age, sex, and BMI. There were 196 individuals who required hospitalization, and 2179 individuals who did not. For this classifier, we did not include any tunable hyper-parameters.

### Making predictions of illness based on health metrics

The physiological signs can be used to predict the onset of COVID-19. Let us denote the *n*^th^ day after the start of symptoms as *D*_*n*_. *D*_0_ is the day when symptoms started. We make the assumption that individuals are healthy, i.e., class “Negative” from day *D*_*a*_ to day *D*_*b*_ where *D*_*b*_ < *D*_0_. Subjects are considered to be sick from day *D*_*c*_ up to day *D*_*d*_ where *D*_*c*_ ≥ *D*_0_. The days between *D*_*b*_ and *D*_*c*_ are treated as a buffer space when subjects may or may not be sick, and hence ignored. The choices of *D*_*a*_, *D*_*b*_, *D*_*c*_, and *D*_*d*_ are made through cross validation. As a guide to choosing the days, we note the median incubation period is estimated at 5.1 days^19^. Since we are using the date relative to the start of symptoms as the ground truth label, we consider only symptomatic individuals.

The following physiological data were calculated for each user on a daily basis using the data recorded from their Fitbit device:

- The estimated mean respiration rate during deep (slow wave) sleep (we default to light sleep if deep sleep data are insufficient).
- The mean nocturnal heart rate during non-rapid eye movement (NREM) sleep.
- The root mean square of successive differences (RMSSD) of the nocturnal RR series.
- The Shannon entropy of the nocturnal RR series.

Data are collected simultaneously from the PPG sensor and the accelerometer. RR data are only stored when no motion above a set threshold is detected, and when the coverage in a 5-min window exceeds 70%. Data are only collected when the subjects are at rest. The RR data are then cleaned to remove noise due to missed heart beats, motion artifacts, electronic noise, etc. The Fitbit system estimates periods of light, deep (slow wave), and REM sleep^20^ and this is used in deciding which sections of the overnight data to process. The respiration rate is obtained by fitting a Gaussian model to the spectrum of the interpolated RR intervals as a function of frequency—this relies on the phenomenon of respiratory sinus arrhythmia (RSA) to induce a measurable modulation of the RR interval series. In cases where there is no discernible RSA, we do not estimate the respiration rate. The RMSSD is a time domain measurement used to estimate vagally mediated changes^8^. It is computed in 5-min intervals, and the median value of these individual measurements over the whole night is calculated. The Shannon entropy is a non-linear time domain measurement computed using the histogram of RR intervals over the entire night. The RMSSD and entropy are computed between midnight and 7 a.m. The sleeping heart rate is estimated from non-REM sleep only. The respiration rate is computed from deep sleep when possible, and from light sleep in the case of insufficient deep sleep.

We validated our respiration rate algorithm against flow sensor data obtained from 19 subjects wearing Fitbit Versa devices. Ground truth data were obtained using polysomnography measurements conducted at the Sleep Med lab in South Carolina. The mean absolute error with 39 nights of data is 0.75 b.p.m. on average with 7 data points greater than 1 b.p.m. error. The large error cases are due to either (i) low signal-to-noise ratio in the RR data (SNR < 3) or (2) individuals with severe apnea. The comparison of respiration rate with the ground truth was done using the whole night’s data. For the COVID-19 detection algorithm, however, we used RR data collected during deep sleep when possible, since sinus arrhythmia is more prominent in deep sleep. Fitbit heart rate and sleep measurements have been studied by an external group^21^ who found that Fitbit Charge HR devices showed a 97% sensitivity and a 91% accuracy in detecting sleep. The average heart rate measured using Fitbit Charge HR devices was 59.3 ± 7.5 b.p.m., while the heart rate measured from ECG was found to be 60.2 ± 7.6 b.p.m.^21^. For details on how Fitbit measures HRV, we refer the reader to Natarajan et al.^22^.

Since health metrics such as respiration rate, heart rate, and HRV can vary substantially between users, we use the *Z* -scored equivalents:

3 $${Z}_{x}=\frac{x-{\mu }_{x}}{{\sigma }_{x}},$$

where *x* could stand for respiration rate, heart rate, RMSSD, or entropy; *μ*_*x*_ and *σ*_*x*_ are the rolling mean and rolling standard deviation of the metric being measured. For each day *D*_*n*_, we construct a 5 × 4 matrix with the normalized *Z*-scores corresponding to the four health metrics, measured on days *D*_*n*_ ⋯ *D*_*n*−4_. Each day is represented by a matrix with that day’s data along with the previous 4 days data. Each row of the matrix represents a day of data, while each column represents a metric. We linearly interpolate missing data, but only do so if there is a minimum of 3 days of data. We create an “image” from each matrix by resizing each 5 × 4 matrix to a 28 × 28 × 1 matrix, with the last dimension indicating that there is only one color channel. The pixel values are rescaled to the range (0,1). We included 1257 symptomatic individuals with sufficient data for analysis; 70% of the subjects were randomly selected to comprise the training set. The remaining 30% of subjects were split equally into two hold-out sets with 15% each: one for cross-validation, and the other for testing. Data present in the test set are considered representative of unseen data, and are reported. This random split of 70:15:15 is performed five times (i.e., five folds), but cross-validation is performed only once.

**Fig. 6:**
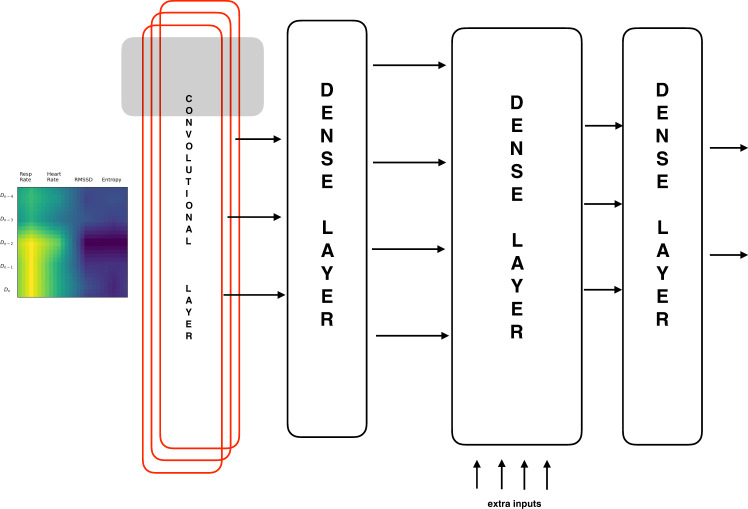
The neural network architecture. The nocturnal respiration rate, heart rate, RMSSD, and entropy for day *D*_*n*_ along with the previous 4 days data are *Z*-scaled, arranged in the form of a 5 × 4 matrix and rescaled to 28 × 28 × 1. This image is fed to a 1-dim. convolutional layer with *m* filters. The first dense layer reduces these *m* features to a smaller number of *N*_1_ features which are concatenated with an array of external inputs such as age, gender, BMI, etc. The last dense layer leads to a softmax filter.

Figure 6 shows the neural network architecture. Each image is input to a 1-dim. convolutional stage with *m* filters, and a filter size of *k*. After maxpooling, the convolutional stage produces a set of *m* features. Non-linearity in the form of a “Relu” layer is introduced. A dense layer is used to reduce the *m* convolutional features to a smaller feature set *N*_1_. At this stage, an array of *n* external inputs is applied including features such as age, gender, and BMI that need to bypass the convolutional stage. In addition, we also include the *Z*-scored respiration rate, heart rate, RMSSD, and entropy for day *D*_*n*_ as a part of the array. The final dense layer leads to a softmax layer with two possible output classes.

### Reporting Summary

Further information on research design is available in the Nature Research Reporting Summary linked to this article.

## Supplementary information

Reporting Summary

## Data Availability

Our consent form and the protocol allow us to make the raw data available to two academic members of our research consortium, namely Stanford University, and Scripps Translational Research Institute. Fitbit’s privacy policy does not permit us to make the raw data available to third parties including researchers outside of our web API Oauth 2.0 consent process. For specific questions, contact Fitbit at https://healthsolutions.fitbit.com/contact/.

## Code availability

The only software used were standard Python packages, and Tensorflow and Keras packages. Fitbit’s intellectual property rules do not permit us to open source the software.
